# Food Security–Climate Change–National Income Nexus: Insights from GCC Countries

**DOI:** 10.3390/foods15061099

**Published:** 2026-03-20

**Authors:** Raga M. Elzaki

**Affiliations:** Department of Agribusiness and Consumer Science, College of Agricultural and Food Sciences, King Faisal University, Al-Ahsa 31982, Saudi Arabia; rmali@kfu.edu.sa

**Keywords:** capital income, carbon emissions, food security status, vulnerability to climate change, Bayesian predictions

## Abstract

Food security is being experienced particularly deeply in vulnerable regions that are impacted by climate change. Therefore, this study aims to examine the impact of climate change and gross national income on food security in the Gulf Cooperation Council (GCC) countries. The study utilized cross-country panel data for GCC countries from 2000 to 2024, with food access acting as the dependent variable for food security. The annual meteorological temperature, energy-related carbon emissions, and gross national income are involved as independent variables representing the factors of climate change and economic growth, respectively. The Pedroni and Johansen–Fisher panel cointegration tests were implemented. Furthermore, the study employs Bayesian random-effects (BRE) and Bayesian mixed-effects (BME) models, estimated through Markov Chain Monte Carlo (MCMC) methods, for achieving posterior distributions of the model’s parameters. The results confirm the existence of a long-term cointegrating relationship among the selected variables. Gross national income has a positive impact on food security, whereas carbon emissions exert a negative effect. The findings reveal that food security is shaped by interconnected economic and climate factors, with notable differences between countries. These results underline the importance of regional cooperation and country-specific policies that focus on enhancing income, mitigating emissions, and investing in food systems.

## 1. Introduction

Food security is a critical policy priority in the twenty-first century, especially in regions vulnerable to climate variability and economic change [1]. It is shaped by a complex set of economic, climate, political, technological, demographic, agricultural, and cultural factors [2].

Climate change represents a fundamental challenge to global economic systems and food security, with increasingly severe negative effects ranging from extreme weather volatility to biodiversity loss, undermining global stability [3]. These environmental shifts translate into thoughtful economic vulnerabilities across key sectors; while the primary sector faces declining yields and resource scarcity that threaten food availability, the industrial sector faces significant supply chain instabilities and rising operational costs [4]. These sectoral disruptions account for a substantial portion of global economic losses attributed to climate-related disasters. For the GCC countries, these impacts are particularly acute given their existing environmental vulnerabilities and high reliance on food imports [5]. Consequently, understanding the complex interactions between climate change, food security, and national income is essential for ensuring long-term economic stability and industrial resilience in the Gulf region.

The GCC countries face dissimilar structural challenges affecting food security, including rapid population growth, high dependence on food imports, limited arable land, water scarcity, and increasing climate risks, which collectively increase the vulnerability of national food systems [6,7]. At the same time, national income, largely driven by hydrocarbon revenues, plays a key role in shaping governments’ ability to invest in food supply chains, adaptive technologies, and climate-resilient infrastructure in these economies [8].

Despite the strategic importance of food security in the GCC, empirical evidence on the joint effects of climate change indicators and national income on food security remains limited. Most existing studies examine these factors separately, overlooking the interconnected nature of climatic stress, economic capacity, and food access [9,10,11]. GCC countries are particularly vulnerable due to extreme temperatures, recurrent droughts, and declining water resources, while their food systems rely heavily on volatile global markets under changing climatic conditions. Simultaneously, fluctuations in national income driven by oil market dynamics may either mitigate or exacerbate food insecurity, depending on fiscal capacity and resilience measures. This highlights the need for a comprehensive, region-specific analysis that captures the dynamic interplay between climate change, national income, and food security in the GCC.

This study examines the impact of climate change and national income on food security in GCC countries, focusing on how temperature variability, carbon emissions, and gross national income affect food access. It further explores cross-country heterogeneity to identify differences in the strength and direction of these relationships across the six GCC economies. Based on the study’s objectives, the following hypotheses are formulated:

**H1:**  *It is hypothesized that food security, climate change, and national income are linked through a long-run cointegration relationship in GCC countries.*

**H2:**  *It is hypothesized that climatic variability exerts statistically significant negative pressure on food security indicators within GCC countries.*

**H3:**  *Higher levels of national income are expected to correlate positively with food security by facilitating increased investment in food imports, resilient infrastructure, and adaptive technologies.*

**H4:**  *The impact of climate change and national income on food security is presumed to vary across GCC states, reflecting differences in their respective economic structures and climatic vulnerabilities.*

By integrating economic and climatic factors, this study provides valuable insights that enable policymakers to design targeted strategies to strengthen food security under changing climatic conditions. Moreover, analyzing the relationship between food security, climate change, and national income is crucial for informing sustainable policy formulation in the GCC context.

Using a Bayesian framework, the study provides probabilistic evidence on the dynamic links among climate variables, income, and food security, supporting the formulation of more informed policy actions. These contributions directly advance several Sustainable Development Goals (SDGs 1, 2, and 13) and indirectly support additional goals (SDGs 8, 12, and 17), thereby promoting sustainable development and enhancing climate resilience. Also, the study can be applied to other regions with similar environmental and economic characteristics, advancing both theoretical understanding and practical policy applications.

## 2. Literature Review

Ensuring food security is a key policy challenge in countries vulnerable to climate stress and economic fluctuations, prompting numerous studies on the joint effects of climatic variability and macroeconomic conditions on food security [12,13,14,15].

### 2.1. Climate Change–Food Security Nexus

Several studies examine the food security–climate nexus across different regions using diverse empirical approaches. Mirón et al. [16] reviews the influence of climate change on food security and suggests that rising carbon dioxide concentrations, together with increasing global temperatures, may theoretically enhance crop yields for human and animal consumption. Moreover, Pickson et al. [17] examined how climate change affects food security in African countries using the Mann–Kendall test, the pooled mean group, and the Dumitrescu–Hurlin panel causality test. The results indicate that rainfall is a key long-term driver of food security, while extreme temperatures primarily negatively affect food security in the short term, with varying effects across countries. Furthermore, Lee et al. [18] examined how extreme and unpredictable temperature changes associated with climate change affect food security. The authors indicated that rising temperatures, coupled with increasing global food prices, exacerbate malnutrition and food insecurity, particularly in developing countries

A study investigated the impact of climate change on food security in China by using regional heterogeneity analysis. The results show that climate change significantly increases food security risks [18]. Another study examined the impact of climate variability on household food security in Tanzania by estimating a panel fixed-effects regression model. The results indicated that low rainfall and cool conditions significantly reduce food security [19]. Furthermore, Mahali et al. [20] investigated the nexus between food security and climatic and non-climatic factors in India using an autoregressive distributed lag (ARDL) model. The findings indicate that carbon emissions significantly influence food security in the short and long run, while temperature has an insignificant short-run effect but a positive long-run impact.

A study examines the relationship between rainfall patterns, temperature variations, and household food security in Ethiopia using ordered logistic regression on household survey data. The results show that increasing temperature leads to high levels of food insecurity [21]. Another study investigates the impact of climate variability on food security in Sub-Saharan African countries by constructing a food security index using Principal Component Analysis and analyzing its relationship with precipitation, temperature, and CO_2_ emissions through the Panel-Corrected Standard Error (PCSE) method. Results indicate that precipitation and CO_2_ emissions positively influence food security, whereas higher temperatures negatively affect it [22].

### 2.2. Income–Food Security Nexus

Recent empirical research consistently shows that gross national income (GNI) is a key determinant of national food security, although the magnitude of the relationship varies across regions and methodological approaches. Another study using a panel fixed-effects model found that higher income levels significantly improve national food security by strengthening agricultural capacity and market participation [23]. Similarly, Nzayiramya et al. [24] applied global demand modeling using a Quadratic Almost Ideal Demand System (QUAIDS). They demonstrated that income strongly influences food consumption patterns, particularly in low-income countries, thus reinforcing the central role of GNI in shaping food access. In contrast, Günal et al. [25], using a multicausality approach with Granger causality and vector error-correction modeling (VECM), found that income growth reduces food insecurity. Additionally, Molotoks et al. [13] used a cross-national regression analysis combined with robustness checks to compare different food security indicators, finding that higher national income improves food security outcomes but does not fully eliminate inequality-driven food access constraints. Further evidence comes from [26], who used structural equation modeling (SEM) to show that socio-economic conditions associated with higher income, such as reduced food loss, improved logistics, and better infrastructure, indirectly enhance food security through more resilient supply chains. The study concluded that, from previous studies, while rising GNI plays a critical and consistent role in strengthening food security, the relationship is facilitated by structural, market, and demographic factors, indicating that income-driven improvements should be complemented by comprehensive policy interventions.

### 2.3. Carbon Emission–Food Security Nexus

Several studies have explored the relationship between carbon emissions and food security across different regions. In Pakistan, Naseem et al. [27] investigated the long-run cointegration between CO_2_ emissions and food security using unit root tests, including ADF, PP, KPSS, and Zivot–Andrews. Their findings indicate an atypical response of CO_2_ emissions to adverse shocks in agricultural value-added, highlighting consequential effects on food security. Similarly, in East Africa, Ntiamoah et al. [28] examined the long-term impact of carbon emissions and macroeconomic variables on food security. Employing heterogeneous panel cointegration tests along with FMOLS and DOLS estimations, the study confirms that both CO_2_ emissions and economic growth positively influence food security. In Nigeria, Fagbemi et al. [29] analyzed the effect of CO_2_ emissions on food production using autoregressive distributed lag (ARDL) and vector error correction mechanism (VECM) models, revealing that carbon emissions are a significant determinant of food production and, consequently, food security. From an environmental and institutional perspective, Rahaman et al. [30] applied Westerlund panel cointegration alongside AMG and PDOLS estimators and found that ICT contributes to improved food security only under strong governance conditions, while CO_2_ emissions and environmental degradation erode long-run food security gains, highlighting persistent climate–economic constraints.

Recent studies have increasingly applied Bayesian methods to analyze food security. Another study employed a Bayesian spatio-temporal model in Africa, identifying persistent hotspots of high food insecurity in the Sahel and Horn of Africa [31]. In Saudi Arabia, Elzaki & Al-Mahish [32] used a Bayesian Vector Autoregression (BVAR) model to show that water shocks exacerbate food insecurity, whereas improved water availability mitigates it. KC et al. [33] applied Bayesian belief networks to Thailand’s Global Food Security Index (GFSI) data, identifying key determinants such as agricultural productivity, market access, and governance, and prioritizing interventions for the most vulnerable regions.

From the reviewed literature, the study concluded that while raised CO_2_ levels may offer limited theoretical yield benefits, climate variability, particularly temperature extremes and rainfall fluctuations, remains a dominant and detrimental driver of food insecurity. While higher income supports food security, it does so only when reinforced by effective policies. Furthermore, in the GCC countries, food security is driven by import dependence, climate stress, population dynamics, governance quality, and supply-chain efficiency. Likewise, the literature review highlights how Bayesian methods can integrate spatial, temporal, and hierarchical information to provide robust, probabilistic insights into food insecurity.

## 3. Materials and Methods

This section outlines the study’s methodological framework, including research design, data sources, variables, and analytical techniques employed. It also describes the diagnostic tests and validation procedures used to ensure the reliability and accuracy of the results.

### 3.1. Data Sources

This study utilizes cross-country panel data for the GCC region spanning 2000 to 2024. Food access (FSAC), proxied by Gross Domestic Product (GDP) per capita at purchasing power parity (constant 2021 international $), serves as the dependent variable. Annual temperature (AMLT) reflects meteorological variations; carbon emissions from energy (COE), representing the environmental footprint of industrial activity; and gross national income (GNI) accounts for the macro-level fiscal strength of the GCC countries. These indicators are interpreted as predictors that represent climate and economic influences. The data was extracted from the FAOSTAT data [34] and from World Development Indicators [35].

Food access is selected as the primary indicator of food security, given the GCC countries’ heavy reliance on imports and limited domestic agricultural capacity. Furthermore, the empirical analysis relies on consistent and comparable long-term panel data across GCC countries. Indicators that adequately examine the other dimensions of food security, such as utilization or stability, are not consistently available for all countries over the study period. While precipitation and extreme weather events are important determinants of food security, this study focuses on long-term thermal and atmospheric drivers, temperature and CO_2_ emissions, because of their sustained impact on agricultural productivity and the greater availability and consistency of long-term data across countries.

Gross national income reflects the economic capacity to secure and stabilize the food supply. In contrast, temperature and carbon emissions exert climate-related pressures that affect food system stability and supply chains. Table 1 presents a summary of descriptive statistics for the selected variables, along with their corresponding units of measurement.

### 3.2. Econometric Estimations

Before estimating the Bayesian models, the study conducted preliminary tests including normality, cross-sectional dependence (CD), homogeneity, unit roots, and panel cointegration. The slope homogeneity and cross-sectional dependence were assessed using the procedure from [36]. Confirming the existence of cross-sectional dependence, panel unit root checks were achieved by applying the Fisher unit root test [37].

Two approaches of panel cointegration tests were implemented: the panel Pedroni [38,39] and Johansen–Fisher [40] tests to govern the presence of a long-term equilibrium relationship among the data series, along with a country cross-section Johansen cointegration test [41]. Therefore, the Pedroni and Johansen–Fisher tests are employed to identify stable, long-run cointegrating vectors between climate shocks, national income, and food security indicators.

#### 3.2.1. Pedroni Panel and Johansen–Fisher Cointegration Tests

The Pedroni test starts with a heterogeneous panel cointegration regression:

(1) $$y_{it}=\alpha_{i}+\delta_{it}+\beta_{i}x_{it}+\varepsilon_{it}$$

This test is divided into two groups: within-dimension (includes 4 panel statistics, pooling the autoregressive term across all cross-sections to test for cointegration in the panel as a whole) and between-dimension (includes 3 group statistics, allowing for heterogeneity in the autoregressive coefficient). This test is based on the Augmented Dickey–Fuller (ADF) test-type regression for the residuals [42].

(2) $${∆e}_{it}=\rho_{i}e_{i,t-1}+\sum_{k=1}^{k}\gamma_{ik}{∆e}_{i,t-k}+u_{it}$$

${∆e}_{it}$ is the first difference of the residual; $e_{i,t-1}$ is thlagged residual; $\rho_{i}$ = is the coefficient on the lagged residual; if $\rho_{i}$ = 0, the residual has a unit root, which means no cointegration (i.e., H0: $\rho_{i}$ = 0); if $\rho_{i}$ < 0, the alternative hypothesis is as follows: (H1) within-dimension: H1: $\rho_{i}$ = ρ < 0 for all *i*; between-dimension: H1: $\rho_{i}$ = ρ < 0 but heterogenous across *i*; *k* = number of lags included; $\gamma_{ik}$ = coefficients for each lag. The study employs model specifications with FSAC as the dependent variable to compute these statistics.

Furthermore, the study employed the Johansen–Fisher panel cointegration test, which combines the Johansen cointegration statistics [43] from each cross-section into a single Fisher-type statistic. Therefore, for a panel with N countries, the Fisher statistics for the trace test and maximum eigenvalue test are

(3) $$Fisher-trace=-2\sum_{i=1}^{N}Ln\left(p_{i}^{trace}\right)\;$$

(4) $$Fisher-MaxEigen=-2\sum_{i=1}^{N}Ln\left(p_{i}^{max}\right)\;$$

where $p_{i}^{trace}$ = *p*-value of the Johansen trace cointegration statistic for cross-section *i*; $p_{i}^{max}$ = *p*-value of the Johansen maximum eigenvalue statistic for cross-section *i*. Both resulting Fisher statistics follow the X^2^ distribution with degrees of freedom = 2N.

The Johansen trace test statistics take the form

(5) $$\mathrm{Trace}\;\mathrm{Statistic}=-T\sum_{i=r+1}^{k}ln(1-{\hat{\lambda }}_{i})$$

where T = number of observations; ${\hat{\lambda }}_{i}$ = estimated eigenvalues; r = number of cointegrating relations under the null hypothesis; and k = total number of variables in the system.

The Johansen maximum eigenvalue test statistic takes the form

(6) $$\mathrm{Max}\text{-}\mathrm{Eigen}\;\mathrm{Statistic}=-T\mathrm{Ln}\left(1-{\hat{\lambda }}_{r+1}\right)$$

where ${\hat{\lambda }}_{r+1}$ = the $\left(r+1\right)$th largest eigenvalue; H0: there are *r* cointegrating vectors; H1: there are *r* + 1 cointegrating vectors.

#### 3.2.2. Bayesian Model

This study employed a Bayesian statistical framework, utilizing Bayes’ theorem [44] to investigate the interrelationships among food security, climate change, and national income. By adopting a Bayesian perspective, this research overcomes the limitations of small-sample panel data (N = 6), providing more reliable posterior estimates and a robust quantification of uncertainty regarding long-term economic stability in the GCC region. This framework manages regional complexity through Bayesian random effects (BRE), which control unobserved heterogeneity by treating differences between GCC countries as random variations; this ensures that national-level heterogeneity does not bias the overall findings regarding income and food security. Additionally, Bayesian mixed effects (BME) are utilized to evaluate both fixed regional trends and random localized variations.

Bayes’ theorem provides a systematic approach for integrating prior knowledge with empirical data, allowing for the continual updating of beliefs about model parameters. Using this methodology, posterior distributions were constructed to represent the updated probabilities for each parameter given the observed evidence. From these posterior distributions, key statistical estimates, posterior means, medians, credible intervals, and Monte Carlo Standard Errors (MCSE) were obtained.

The reduced form of Bayesian takes as a proportion

(7) Prδyv=PryvδPrδPryv

yv represents the variables under investigation, which include food access, meteorological temperature, carbon emissions, and national income. δs represents unknown parameters. Pr(*yv*|δ) represents the likelihood function, which is the probability of *y* assuming δ. Pr(δ) is the prior distribution of δ. Pr(*yv*) is the marginal distribution of *y*, and Pr(δ|*yv*) is the posterior distribution, which is the probability of δ provided y.

This study, following Lemoine [45], assumes a normal distribution N(0,1) for the observed variables and a Gamma distribution (0.01, 0.01) for the variances (σ^2^) within the Bayesian model. Accordingly, the prior distributions were specified as θ ∼ N(0, 1) and σ^2^ ∼ Gamma(0.01, 0.01) and chosen as weakly informative priors. These specifications help stabilize estimation in relatively small samples while avoiding strong prior assumptions that could dominate the likelihood.

For estimation, the study adopted the BRE regression framework outlined by Mai [46], utilizing Metropolis–Hastings and Gibbs sampling techniques within a Markov Chain Monte Carlo (MCMC) simulation. The likelihood function is assumed to follow a normal distribution, conditional on the variance parameter σ^2^, allowing us to estimate the latent structure and uncertainty in the model related to food access, meteorological temperature, carbon emissions, and gross national income.

A basic BRE assumes that each cross-section (or group) i has its own intercept (or parameter) drawn from a common prior distribution.

(8) $$y_{it}=\alpha_{i}+\beta x_{it}+\varepsilon_{it},\alpha_{i}∼N(\mu_{\alpha },\tau_{\alpha }^{2})$$

The model structure involved likelihood (BRE), which takes the form:

(9) $$y_{it}=\alpha_{i}+\beta x_{it}+\varepsilon_{it},\varepsilon_{it}\sim N(0,\sigma^{2})$$

and RE, which takes the form:

$\alpha_{i}∼N\left(\mu_{\alpha },\tau_{\alpha }^{2}\right);$ as well as the prior distributions, which take the form:

$$\mu_{\alpha }∼N(0,\sigma_{\mu }^{2}),\;\tau_{\alpha }^{2}∼\mathrm{Inv}\text{-}\mathrm{Gamma}(a_{\tau },b_{\tau })\;\beta ∼N(0,\sigma_{\beta }^{2}),\;\sigma^{2}∼\mathrm{Inv}\text{-}\mathrm{Gamma}(a_{\sigma },b_{\sigma })$$

The posterior distributions take the form:

(10) $$p(\alpha_{i},\mu_{\alpha },\beta ,\sigma^{2},\tau_{\alpha }^{2}|y)\propto \prod_{i,t}p(y_{it}|\alpha_{i},\beta ,\sigma^{2})\;p(\alpha_{i}|\mu_{\alpha },\tau_{\alpha }^{2})\;p(\mu_{\alpha })p(\beta )p(\sigma^{2})p(\tau_{\alpha }^{2})$$

where N(μα, $\tau_{\alpha }^{2}$) is a normal distribution with mean *μα* and variance $\tau_{\alpha }^{2}$. $\sigma_{\mu }^{2}$ is the prior variance for the mean of random effects; $\sigma_{\beta }^{2}$ is prior variance for the fixed-effect coefficient; $a_{\tau },b_{\tau }$ represent the parameters of the inverse-gamma prior for the random-effect variance $\tau_{\alpha }^{2}$ and $a_{\sigma },b_{\sigma }$ stands for parameters for the prior on the error variance $\sigma^{2}$. Moreover, the study examined the BME (hierarchical) model, which allows random intercepts and slopes, in addition to fixed effects.

The BME equation takes the form:

(11) $$y_{it}=x_{it}^{\prime }\beta +z_{it}^{\prime }u_{i}+\varepsilon_{it},u_{i}∼N\left(0,D\right)$$

The BME model structure involved the likelihood:

(12) $$y_{it}=x_{it}^{\prime }\beta +z_{it}^{\prime }u_{i}+\varepsilon_{it}$$

where $x_{it}$ = fixed-effect covariates; β = fixed-effect coefficients; $z_{it}$ = covariates for random effects; $u_{i}$ = random-effects vector for group i and $\varepsilon_{it}∼N(0,\sigma^{2})$. In addition, the random effects distribution is $u_{i}∼N(0,D)$, where D is a covariance matrix and takes the form:

(13) $$D=\left(\begin{array}{cc} \tau_{1}^{2} & \rho \tau_{1}\tau_{2}\\ \rho \tau_{1}\tau_{2} & \tau_{2}^{2} \end{array}\right)$$

for random intercept and slope. Likewise, the prior distributions are $\beta ∼N(0,\sigma_{\beta }^{2}I)$; $\sigma^{2}∼\mathrm{Inv}\text{-}\mathrm{Gamma}\left(a_{\sigma },b_{\sigma }\right)$ and D∼Inverse-Wishart(ν,S). Posterior distributions take the form:

(14) $$p(\beta ,u_{i},\sigma^{2},D|y)\propto \prod_{i,t}p(y_{it}|\beta ,u_{i},\sigma^{2})\;p(u_{i}|D)\;p(\beta )\;p(\sigma^{2})\;p(D)$$

The BRE framework is adopted because global food security data are complex and often unbalanced. Unlike classical panel methods, the Bayesian approach provides probabilistic estimates, better examines parameter uncertainty through posterior distributions, and accounts for cross-country differences, making the estimated effects more robust to extreme observations and regional heterogeneity.

Additionally, Bayesian diagnostic tests were used, including visual checks for MCMC convergence (Gelman–Rubin), posterior predictive checks, and an assessment of the efficiency of Bayesian estimation.

The 15-year posterior predictions for LogFSAC are generated via a conditional predictive simulation framework. Because cointegration confirms a stable long-run relationship, this static specification is designed to investigate the equilibrium state of the nexus. Unlike recursive dynamic models, this approach integrates the likelihood of future observations over the joint posterior distribution of the parameters, conditioned on the projected paths of AMLT, COE, and GNI. This method is econometrically robust for small panels because it avoids the biases associated with dynamic terms while remaining anchored to long-run elasticities. Consequently, these results represent scenario-based structural projections rather than stochastic short-term forecasts.

#### 3.2.3. PCSE and FGLS Approaches

Because the study detected autocorrelation and heteroskedasticity in the data, the Panel-Corrected Standard Errors (PCSE) and Feasible Generalized Least Squares (FGLS) are used as robustness checks to confirm that the relationships found in the Bayesian model remain consistent under alternative frequentist methods. While the Bayesian model accounts for cross-country heterogeneity and provides probabilistic estimates, PCSE and FGLS address heteroskedasticity and serial correlation, ensuring the results are stable and reliable. The PCSE method is utilized to address potential cross-sectional dependence and spatial correlation inherent in the interconnected GCC economies, thereby providing more accurate statistical inference. Concurrently, the FGLS estimator is implemented to mitigate issues of heteroscedasticity and serial correlation within the panel data. By integrating these frequentist robust estimators alongside the Bayesian framework, the study achieves a comprehensive validation of the long-term relationships between climatic drivers, national income, and food security indicators.

The PCSE approach addresses first-order autocorrelation by utilizing the transformation matrix developed by Prais and Winsten [47]. The basic form of the PCSE estimation equation is as follows:

(15) $${LnFSAC}_{it}=\alpha +\varphi_{1}\mu {LnAMLT}_{it}+\varphi_{2}{LnCOE}_{it}+\varphi_{3}{LnGNI}_{it}+\sum_{i=1}^{i}\delta_{t}D_{t}+\pi_{it}$$

${LnFSAC}_{it}$ represents the natural logarithm of food access, subscript *i* stands for the selected GCC countries, and t specifies the time from 1990 to 2024. α is the intercept term. $\varphi_{1}$, $\varphi_{2}$, and $\varphi_{13}$ are the slopes of the respective temperature, carbon emissions, and gross national income in the model, and $\pi_{it}$ denotes the model’s error stochastic term (idiosyncratic error term). *i* = 1,. …; N; *t* = 1, …; T.

$D_{t}$ represents time dummies (a dummy for each period t, with one omitted for collinearity), and $\varepsilon_{it}$ represents the errors.

The FGLS estimator is employed to address panel data characterized by heteroskedasticity and contemporaneously correlated error terms, following Parks [48]. The general form of the FGLS model is expressed as:

(16) $${FSAC}_{it}=\alpha +\beta_{1}\mu {LnAMLT}_{it}+\beta_{2}{LnCOE}_{it}+\beta_{3}{LnGNI}_{it}+\varepsilon_{it}$$

$\varepsilon_{it}$ denotes the idiosyncratic error term, which reflects all unobserved factors affecting FSAC that are not included in the study model and are often characterized by heteroskedasticity and contemporaneously correlated error terms.

## 4. Results

### 4.1. Preliminary Results

#### 4.1.1. Normality Results

Table 2 presents the results of normality tests for the study’s variables. The results indicate that the selected variables do not follow a normal distribution, as evidenced by significant skewness and/or kurtosis and JB tests. Therefore, before conducting further analyses, it is suggested to transform them using their natural logarithms (log). National income was transformed in log–log form to allow elasticity interpretation and to address its highly skewed distribution.

#### 4.1.2. Cross-Sectional Dependence and Slope Homogeneity Results

The Pesaran CD test results indicate strong cross-sectional dependence among most variables in the panel, with significant CD statistics and *p*-values of 0.00 for LogAMLT, LogCOE, and LogLogGIN, and high mean correlation coefficients ranging from 0.87 to 0.98 (Table 3). This highlights that shocks or policy changes in one GCC country are likely to impact others, reflecting the interconnected economic, climate, and social dynamics within the region. In contrast, LogFSAC displays insignificant cross-sectional dependence (*p* = 0.055), suggesting relatively independent variation in food security across countries. Notably, the negative mean correlation coefficient (−1.917) for LogFSAC implies that increases in food security in one country may be associated with decreases in another. These findings underscore the importance of using estimation techniques that account for cross-sectional dependence to ensure robust and unbiased results. In this context, Bayesian models are particularly effective, as they can explicitly account for and model the interdependencies among GCC countries.

The Hashem Pesaran and Yamagata [36] slope homogeneity test was applied to assess whether the coefficients of the independent variables are identical across the panel units (GCC countries). The results show that the Delta statistics and adjusted Delta for FSAC are 7.242 and 8.307, respectively (Table 4). Consequently, the H0 of slope homogeneity is rejected, indicating substantial slope heterogeneity and suggesting that the effects of the explanatory variables differ across countries. Given this heterogeneity, models that allow for varying slopes are more appropriate. Accordingly, a Bayesian hierarchical model was employed.

#### 4.1.3. Unit Root Results

The results from the Fisher-type unit root tests with a time trend reveal that, in their level forms, the three variables, namely LogFSAC, LogCOE, and LogLogGIN, are non-stationary, as indicated by high *p*-values for the P, Z, L*, and Pm statistics (Table 5). This means the null hypothesis of a unit root cannot be rejected for these variables. The exception is LogAMLT, which is found to be stationary at levels, with all test statistics significant at the 1% level. After applying the first difference, all variables display strong evidence of stationarity, with all four test statistics highly significant (*p*-values = 0.00).

### 4.2. Cointegration Results

#### 4.2.1. Pedroni Residual Cointegration Test

Table 6 reports the Pedroni residual-based cointegration test results for the LogFSAC model with a deterministic intercept and trend. The within-dimension Panel PP (−2.1471, *p* < 0.05) and Panel ADF (−3.3726, *p* < 0.01) statistics reject the null hypothesis of no cointegration. Consistent evidence is also obtained from the between-dimension Group PP (−1.5851, *p* < 0.10) and Group ADF (−1.7234, *p* < 0.05) statistics. Although the v- and rho-statistics are not significant, the PP and ADF tests, considered the most robust in Pedroni’s framework, confirm the existence of a long-run cointegrating relationship among the variables.

#### 4.2.2. Johansen–Fisher Panel Cointegration Test

The Johansen–Fisher panel cointegration test provides robust evidence of long-run relationships among LogFSAC, LogAMLT, LogCOE, and LogLogGIN in GCC countries (Table 6). At the panel level, the null hypothesis of no cointegration is strongly rejected by both the trace and maximum eigenvalue Fisher statistics (trace = 62.42, *p* < 0.01; max-eigen = 33.91, *p* < 0.01). The hypothesis of at most one cointegrating vector is also rejected (trace = 35.96, *p* < 0.01; max-eigen = 21.45, *p* < 0.05), while the null hypotheses of at most two and at most three cointegrating vectors are not rejected, indicating the presence of two cointegrating relationships in the panel.

The cross-sectional, country-level results further confirm this finding. In the absence of cointegration (r = 0), three GCC countries display statistically significant trace statistics at the 1% level, notably Bahrain (80.32), Qatar (78.90), and the UAE (72.46). The maximum eigenvalue test also offers moderate support for cointegration, particularly in the case of Qatar (35.14). However, at higher cointegration ranks (r ≤ 2 and r ≤ 3), most *p*-values exceed the 5% significance threshold. For example, the UAE records a trace statistic of 17.70 (*p* = 0.36), while Saudi Arabia reports a trace statistic of 17.19 (*p* = 0.40), indicating the absence of additional long-run cointegrating relationships at the individual country level (Table 6).

Following the confirmation of panel cointegration, the Bayesian models are specified in levels to directly identify the long-run equilibrium parameters. While Error Correction Models (ECMs) examine the short-run adjustments, a static specification in levels provides asymptotically consistent estimates of the long-run cointegrating vector [50]. This approach is preferred here to preserve degrees of freedom, given the small cross-sectional dimension (N = 6).

### 4.3. Results of the Bayesian Approach

#### 4.3.1. Results of the BRE Model

Table 7 presents the posterior summaries from the BRE model for food security access. The posterior distributions are generally symmetric, as evidenced by the close alignment of each parameter’s mean and median. The analysis reveals that LogCOE has a significant negative impact on food security access (mean = −0.3777, 95% with a credible interval ranging from −0.4933 to −0.2616), whereas LogLogGNI exhibits a strong positive effect (mean = 5.3323, with a credible interval ranging from 3.7975 to 6.8636). In contrast, LogAMLT is not statistically significant (mean = −0.0212, with a credible interval ranging from −0.0493 to 0.0068). The insignificance of temperature likely reflects the GCC’s arid climate and adaptive measures, such as irrigation and desalination, which reduced its short-term impact on food security compared to factors like income and emissions.

All coefficient MCSE values are very low and close to zero, indicating high precision and strong MCMC convergence. The model also demonstrates good sampling performance, with an 84% acceptance rate and efficiency rate ranging from 1% to 72.73%. The estimated random-effects variance (var_U = 0.05736) reflects notable differences across countries, while the small residual variance (sigma^2^ = 0.00176) suggests limited unexplained variation. These results indicate that higher gross national income improves food access, higher carbon emissions reduce it, and temperature has no significant effect.

#### 4.3.2. Results of the BME Model

Furthermore, the estimation of a BME (hierarchical) model was investigated, which incorporates both fixed and random effects (Table 8). The model results show that LogAMLT has a negative but statistically insignificant effect on food security access (β = −0.0167, *p* < 0.12), indicating that higher temperatures tend to reduce food security access, although the effect is not strong enough to be conclusive. LogCOE exhibits a significant negative impact (β = −0.3399, *p* < 0.001), implying that climate degradation is strongly associated with lower food security access across countries. In contrast, LogLogGNI has a highly significant positive effect (β = 4.0785, *p* < 0.001), suggesting that higher national income improved food security outcomes. The random-effects estimates reveal substantial between-country variability, particularly in income (var = 2.3346) and the intercept (var = 1.6427). The very high conditional intraclass correlation coefficient (ICC = 0.9995) indicates that most of the variation in food security access is explained by country-level differences. Furthermore, the inclusion of a random-effects structure substantially improves the model fit, as evidenced by χ^2^ (6) = 402.00; the log-likelihood value of 284.25892 provides a useful benchmark for comparing this specification with alternative models.

The observed high conditional intraclass correlation coefficient (ICC = 0.9995) suggests that nearly all variations in food security access are attributable to persistent differences between countries rather than annual fluctuations. Substantially, this reflects the genuine structural heterogeneity within the GCC countries. Although these nations share a geographic region and trade framework, their baseline levels of food security are heavily determined by country-specific factors such as national wealth (GNI per capita), domestic infrastructure, and population scale.

#### 4.3.3. Results of the Posterior Summary Model

The posterior summary statistics provide insights into the variability and central tendency of food security access (FSAC) across GCC countries, highlighting disparities and uncertainties (Table 9). The results highlight differences in food security access (FSAC) among GCC countries, reflecting regional differences in economic structures, food systems, and policy priorities. Qatar shows a strong positive association (mean = 0.2680, 95% CI: [0.1368, 0.3995]). This result may be related to the country’s high GDP per capita, well-developed food import systems, and ongoing investments in food security initiatives, including aquaculture projects and food storage infrastructure. Similarly, Saudi Arabia (mean = 0.1082) and the UAE (mean = 0.1115) also exhibit positive associations. These outcomes may reflect the role of strong economic capacity, diversification strategies, and government-led food security programs, such as the UAE’s National Food Security Strategy, which aims to strengthen food supply resilience.

In contrast, Oman shows the most pronounced negative association (mean = −0.2010, 95% CI: [−0.3326, −0.0686]). This pattern may be associated with structural constraints such as limited agricultural capacity, reliance on food imports, and exposure to fluctuations in global food prices. Bahrain (mean = −0.0874) also displays a modest negative association, which could be related to structural characteristics such as limited land availability and dependence on external food supply. Kuwait’s near-neutral association (mean = −0.0032) suggests a relatively balanced outcome, possibly reflecting the coexistence of strong financial resources alongside structural limitations in domestic food production.

The low residual variance in FSAC (e.LogFSAC: sigma2 = 0.0018) indicates the robustness of the model, suggesting most variability is explained by country-level differences. These findings emphasize the importance of personalized policies to address unique challenges: Qatar’s success highlights the value of positive investments, while Oman and Bahrain may need targeted strategies to mitigate vulnerabilities in food import dependence and enhance local food systems.

### 4.4. Diagnostics of Bayesian Estimation for Food Security Access

Bayesian diagnostic tests were conducted to ensure convergence of the MCMC chains, assess sampling efficiency, and confirm the reliability and robustness of the optimal posterior distribution. Bayesian model convergence was evaluated using both graphical diagnostics and the Gelman–Rubin statistic, based on five parallel chains estimated under default prior specifications.

#### 4.4.1. Visual Diagnostics for MCMC Convergence

**Figure 1:**
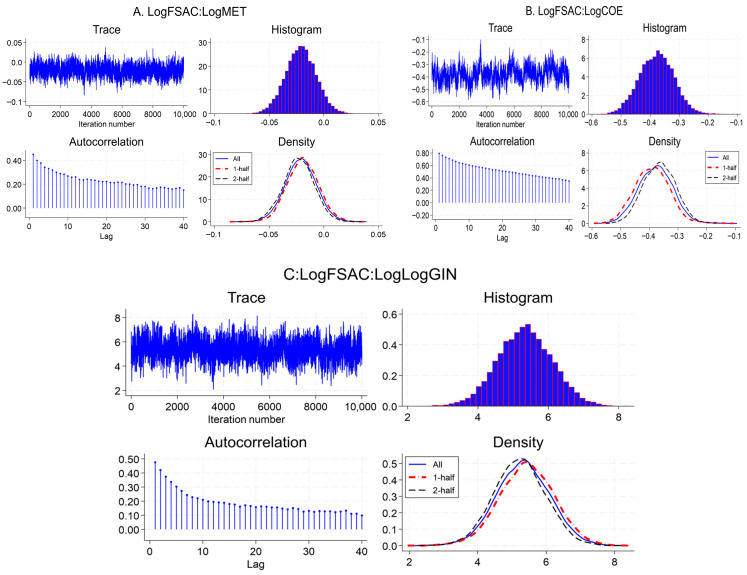
MCMC diagnostics for FSAC and predictor variables. Note: The curve confirms convergence and reliability: the trace plot shows stable mixing, the histogram indicates a normal-like distribution, the autocorrelation plot reveals efficient sampling with decreasing dependency, and the density plot demonstrates consistency across the full chain and its halves.

The trace plots for LogMET, LogCOE, and LogLogGIN fluctuate around stable means with no visible trends, indicating good mixing and convergence. Autocorrelation declines steadily for all variables, dropping below 0.2 by lag 15, suggesting efficient sampling. The histograms display smooth, unimodal, and approximately normal posterior distributions for each predictor. Density plots for the full chain and split samples closely overlap, further confirming stability. Therefore, the Bayesian model for LogFSAC effectively integrates prior information with the likelihood of the data, and the diagnostics confirm convergence and stability across predictors, as illustrated in Figure 1. These findings demonstrate that the MCMC chains have converged and that the BME model provides reliable and robust posterior estimates for the LogFSAC predictors.

#### 4.4.2. Gelman–Rubin Convergence (RC) Statistic

The diagnostics confirm that the Bayesian estimation of the FSAC model has achieved satisfactory convergence. As shown in Table 10, all parameters meet the convergence criteria, with RC values close to 1.0, indicating well-mixed MCMC chains sampled from the same posterior distribution. Table 10 highlights that RC values for FSAC predictors, LogLogGIN (1.001659), LogCOE (1.001355), and LogAMLT (1.000879) as well as the intercept (_cons = 1.002009) and residual variance (sigma2 = 1.000853), are all below the threshold of 1.1. These results confirm the stability and reliability of the posterior estimates, ensuring the robustness of the model’s Bayesian inference.

#### 4.4.3. Posterior Distributions of Panel Effects

**Figure 2:**
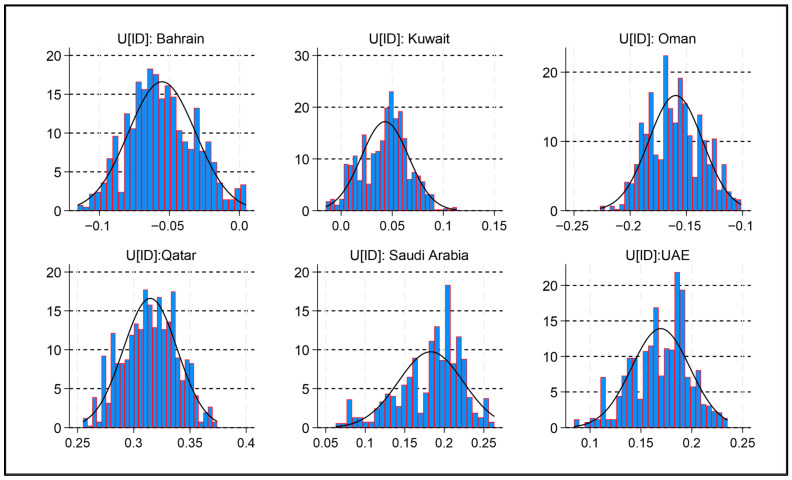
Posterior distributions of country-level random effects (U[ID]) in the FSAC model. Note: *X*-axis represents the posterior value of random effect (U[ID]) and *Y*-axis represents posterior sample count (frequency).

Figure 2 illustartes the histograms of the posterior distributions of the country-specific random intercepts (U[ID]) from the Bayesian mixed-effects FSAC model for the six GCC countries. Each distribution shows the spread and central tendency of the estimated random effects. Bahrain’s effect ranges approximately from −0.10 to 0.00, with a posterior mean near −0.05, indicating a slight negative deviation from the regional baseline. Kuwait shows values from about 0.00 to 0.12, centered around 0.06, reflecting a modest positive effect. Oman exhibits a specifically negative effect, ranging from −0.25 to −0.10, with a mean of −0.17. Qatar has a positive distribution between 0.25 and 0.40, centered around 0.32, while Saudi Arabia shows values from 0.05 to 0.25, with a mean of approximately 0.15. Finally, the UAE displays a positive effect ranging from 0.10 to 0.25, centered near 0.18. The approximately normal shapes of all curves confirm that the model’s assumption of normally distributed random effects is appropriate and that the MCMC sampling achieved stable and well-mixed posterior estimates.

#### 4.4.4. Bayesian Posterior Predictions

The Bayesian posterior predictions for LogFSAC over a 15-step forecast horizon indicate a stable, consistent pattern in anticipated food security access levels (Table 11). Across the horizons for prediction horizons 1 through 13, the posterior means (pmean) remain tightly clustered between 4.69 and 4.76, suggesting no abrupt shifts in projected outcomes. The observed LogFSAC values show close alignment with these posterior means, reinforcing the model’s strong predictive performance. Moreover, the 95% credible intervals are relatively narrow, ranging approximately from 4.61 to 4.85, demonstrating a high degree of certainty around the forecasts. For instance, at horizon 1, the posterior mean is 4.692 with a credible interval of 4.613 to 4.785, while at horizon 15, the mean is 4.741 with bounds between 4.656 and 4.822. This consistency across all 15 prediction points indicates that the Bayesian model provides robust and reliable forecasts of food security access without evidence of increasing uncertainty or divergence over time.

The posterior predictive summary provides an additional assessment of model fit by comparing the distribution of replicated data with the observed statistics (Table 12). The results show that the posterior predictive mean for the minimum predicted LogFSAC (pmin) is 4.5168, with a standard deviation of 0.0246, while the expected observed value E(T_obs) is 4.5641. The corresponding posterior predictive *p*-value, P(T ≥ T_obs) = 0.02, is relatively low, indicating that the observed minimum lies near the tail of the predictive distribution and may signal slight underestimation in this lower range. In contrast, the maximum predicted LogFSAC (pmax) has a posterior mean of 5.1576 (SD = 0.0259), with E(T_obs) = 5.1631 and a predictive *p*-value of 0.38, suggesting a good alignment between predicted and observed maxima.

#### 4.4.5. Efficiency Assessment of Bayesian Estimation in the FSAC Model

The Bayesian efficiency diagnostics indicate successful MCMC convergence, with sampling efficiency varying across parameters. The findings from Table 13 depict that effective sample sizes (ESS) for the predictors show modest efficiency and some autocorrelation, as reflected in ESS ranging from 17.02 (for logCOE) to 44.51 (for loglogGIN) and efficiency values of 0.0365 (logMET), 0.0170 (logCOE), and 0.0445 (LogLogGIN), associated with correlation times of 27.39, 58.76, and 22.47, respectively. In contrast, the residual variance parameter (σ^2^) and variance of the random effect (var_U) show very high efficiency (0.8357 and 1.0000, respectively) and large ESS (835.68 and 1000.00, respectively), indicating highly effective sampling for these parameters. The efficiency values indicate that, despite autocorrelation in some predictors, the posterior estimates are reliable and well-supported by the MCMC sample.

### 4.5. Robustness Checks

The results of the PCSE and FGLS estimators for food security access indicate that carbon emissions and national income are significant determinants, while meteorological temperature is not significant. Specifically, LogCOE has a negative and significant effect on food security access (PCSE: β = −0.189, SE = 0.076, Z = −2.49, *p*-value = 0.013; FGLS: β = −0.189, SE = 0.090, Z = −2.10, *p*-value = 0.035), suggesting that higher carbon emissions are associated with lower food security access. In contrast, LogLogGIN has a positive and significant impact (PCSE: β = 5.084, SE = 1.664, Z = 3.06, *p*-value = 0.002; FGLS: β = 5.084, SE = 1.878, Z = 2.71, *p*-value = 0.007), indicating that higher national income improves food security access. LogAMLT is negative but not statistically significant (PCSE: β = −0.011, *p*-value = 0.597; FGLS: β = −0.011, *p*-value = 0.753), implying that variations in meteorological temperature do not meaningfully influence food security access. Both models are statistically robust, as indicated by the Wald statistics (PCSE: X^2^ = 52.91, *p*-value < 0.01; FGLS: X^2^ = 10.10, *p*-value = 0.0178), with no autocorrelation (Table 14).

## 5. Discussion

This study provides robust empirical evidence on the economic and climate determinants of food security access in the GCC, highlighting substantial cross-country heterogeneity and the importance of country-specific dynamics. The rejection of slope homogeneity confirms that food security drivers vary across GCC countries, justifying the use of Bayesian hierarchical and mixed-effects models and aligning with earlier evidence of regional differences [10,32,51]. The results identify a stable long-run cointegrating relationship among food security access, gross national income, carbon emissions, and temperature. The long-run cointegration results of the study variables also find significance in the Canadian context, where [52] identified climate impacts on the food system as critical determinants of human health risks.

Bayesian estimates consistently show that gross national income has a strong and statistically significant positive effect on food security access, underscoring the central role of economic capacity in an import-dependent region [53,54]. The finding that GNI acts as a primary stabilizer for food access is consistent with other high-income, import-dependent regions like Singapore and Hong Kong, where fiscal capacity serves as a vital buffer against global supply chain shocks [55]. However, the study results suggest a different structural vulnerability compared to global reviews by [56], which emphasizes that climate change threatens food security primarily through biodiversity loss and the degradation of rain-fed agriculture in agrarian regions. Beyond these macroeconomic stabilizers, the literature increasingly highlights the role of agroecology as a transdisciplinary solution to climate-induced food insecurity, as noted by [57]. In contrast, carbon emissions have a significant negative impact, reflecting the adverse effects of environmental degradation and climate-related pressures on food systems, consistent with [58]. These findings highlight the importance of emissions reduction and investment in low-carbon technologies. Meteorological temperature does not emerge as a significant determinant, suggesting that its effects are mitigated by adaptation mechanisms such as food imports, infrastructure, and policy interventions in highly import-dependent economies [59]. However, a study in Asia [60,61] demonstrated that increasing temperatures dramatically reduce rice yields. This creates dual vulnerability for the GCC countries. While the study results show that temperature has no significant impact on food security, the Asian context reveals a simultaneous threat to the global supply side, suggesting that the GCC countries may face diminishing returns if global yield stability continues to decline.

Country-specific results reveal marked differences in food security access across the GCC. Qatar, Saudi Arabia, and the UAE demonstrate relatively strong performance due to higher income levels, diversified economies, and proactive food security strategies, while Oman and Bahrain face greater vulnerabilities linked to structural constraints and limited fiscal capacity, with Kuwait remaining near the regional average. These outlines are supported by prior studies emphasizing technology investment, strategic reserves, and excellent wealth funds in stronger-performing countries [62,63] and fiscal limitations in smaller economies [64].

Robustness checks using PCSE and FGLS estimators corroborate the Bayesian findings, confirming the positive role of income and the negative impact of carbon emissions, while temperature remains statistically insignificant. Generally, the results support the need for differentiated, country-specific food security policies across the GCC, as emphasized by [65]. GCC wealthier countries are focusing on sustainability and emissions reduction, and more vulnerable economies are benefiting from targeted financial and technological support [66,67].

## 6. Conclusions

The nations of the Gulf Cooperation Council (GCC) face a distinct set of structural challenges that significantly impact their food security. Rapid population growth, heavy reliance on imported food, limited cultivable land, water scarcity, and intensifying climate threats have increased the vulnerability of their domestic food systems. Therefore, this study aims to examine how climate change and gross national income affect food security in the GCC region, using panel data spanning the period from 2000 to 2024. To achieve the study’s objectives, the appropriate data were collected, and preliminary econometric estimations were applied. Pretests for cointegration were conducted utilizing the advanced Pedroni and Johansen–Fisher panel cointegration tests. The analysis then employed Bayesian random-effects and Bayesian mixed-effects models, estimated via Markov Chain Monte Carlo (MCMC) methods, to obtain posterior distributions of the model parameters. Additionally, robust checks were performed using the PCSE and FGLS estimators.

The results show that food security access in GCC countries is shaped by strong interconnections among key economic and climate variables but also reveal significant country-specific variation. The results show that determinants such as gross national income and carbon emissions have significant positive and negative effects, respectively, while temperature does not significantly influence food security access. The results reveal that long-run cointegration tests confirm a persistent equilibrium across countries, and Bayesian random- and mixed-effects models show substantial differences between countries, with high ICC values highlighting the role of national context. Posterior analyses reveal differences among countries, with Qatar, Saudi Arabia, and the UAE performing better due to strong economic resources and proactive policies, while Oman and Bahrain face vulnerabilities. MCMC diagnostics, Gelman–Rubin statistics, efficiency measures, and 15-step forecasts show the robustness, stability, and predictive reliability of the Bayesian estimates. Complementary PCSE and FGLS results reveal that income and carbon emissions are robust determinants of food security.

## 7. Policy Implications, Recommendations and Limitations

The results revealed that food security in GCC countries requires a combination of long-term, structural, and country-specific policies. While regional cooperation is important due to economic and climate interconnectedness, most interventions should be adjusted to individual country contexts, reflecting cross-country heterogeneity in food security access drivers. Policies should prioritize boosting national income, reducing carbon emissions, and making positive investments in food systems, with wealthier nations providing models for effective strategies. The robustness and convergence of Bayesian model estimates emphasize the credibility of these evidence-based policy recommendations, supporting informed decision-making and reliable planning for sustainable food security across the region.

Based on these results, the study recommends adopting both region-wide and country-specific policies that enhance income growth, reduce carbon emissions, and strengthen food systems, while accounting for cross-country heterogeneity in economic and climate conditions affecting food security.

The study recommends that future research should incorporate a broader range of socio-economic and other factors, such as water scarcity, technological adoption, policy interventions, and trade policies, to better understand their impact on food security in GCC countries. In addition, analyzing food security at the subpopulation level would provide valuable insights, enhance understanding of food insecurity beyond national averages, and help policymakers design targeted interventions for groups that may face higher risks.

Furthermore, examining local adaptation and global supply chain vulnerabilities can provide a deeper understanding of GCC food security and help policymakers design strategies that are resilient to both long-term climate change and short-term external shocks such as disruptions in international trade caused by conflicts, pandemics, or sudden economic crises.

This study has several limitations that should be acknowledged. First, the cross-sectional dimension is limited to the six member states of the GCC (N = 6). While the 25-year time series (T = 24) provides sufficient observations for long-run cointegration analysis, the small number of countries limits the breadth of the hierarchical random slopes. Although Bayesian hierarchical modeling was selected specifically for its ability to provide stable estimates in small populations through partial pooling, the results should be viewed as high-validity insights for the GCC countries rather than universal generalizations for all arid regions. Second, GDP per capita (food access) is used as a proxy for economic access to food. Still, it does not fully capture intra-household distribution or other dimensions of food security, such as nutritional quality, food utilization, food stability, and food availability. Third, the study relies primarily on average temperature and emissions as indicators of climate change, thereby overlooking the potential effects of other climate factors, such as extreme weather events and precipitation variability. Finally, the use of macro-level indicators may not fully reflect the influence of localized technological or policy interventions that could affect food security outcomes at the micro or sectoral level in GCC countries.

## Figures and Tables

**Table 1:** Descriptive analysis of GCC countries’ panel data.

Variable	Units	Statistic	Mean	Std. Dev.	Min	Max	Observations
FSAC	U.S. dollars	Overall	67,486.24	24,832.17	36,654.2	145,591	N = 150
		Between		25,517.4	41,374.27	114,439.6	n = 6
		Within		8389.309	45,162.43	98,637.63	T = 25
AMLT	Degrees Celsius	Overall	1.493073	0.486	0.557	2.722	N = 150
		Between		0.233	1.19776	1.746	n = 6
		Within		0.436	0.474	2.469	T = 25
COE	Kilotons (kt).	Overall	156,043.4	179,585.7	15,810.48	742,060.5	N = 150
		Between		183,148.6	27,757.54	515,389.6	n = 6
		Within		64,114.04	−72,241.32	382,714.4	T = 25
GNI	U.S. dollars	Overall	2.19 × 10^11^	2.60 × 10^11^	8.65 × 10^9^	1.25 × 10^12^	N = 150
		Between		2.33 × 10^11^	2.70 × 10^10^	6.48 × 10^11^	n = 6
		Within		1.48 × 10^11^	−2.45 × 10^11^	8.19 × 10^11^	T = 25

Note: FSAC = food access; AMLT = annual meteorological temperature; COE = carbon emissions from energy; and GNI = gross national income. Between: represents variation among countries/entities (n = 6). Within: represents variation within each country/entity over time (T = 25). Source: author’s calculations based on the collected data (2026).

**Table 2:** Skewness and kurtosis tests for normality.

Variable	Pr (Skewness)	Pr (Kurtosis)	Joint Test	Jarque–Bera X^2^ (*p*-Value)	Normality Status
			Adj X^2^ (*p*-Value)		
FSAC	0.000 ***	0.067 *	23.91 (0.00) ***	41.72 (0.000) ***	Not normal
AMLT	0.077 *	0.009 ***	8.83 (0.012) **	6.252 (0.044) **	Not normal
COE	0.000 ***	0.000 ***	43.87 (0.000) ***	121.1(0.000) ***	Not normal
GNI	0.000 ***	0.000 ***	53.69 (0.000) ***	210.8 (0.000) ***	Not normal

Note: ***, **, and * represent statistical significance at the 1%, 5%, and 10% levels, respectively. Source: author’s calculations (2026).

**Table 3:** Pesaran CD test.

Variable	CD-Test	*p*-Value	Average Joint T	Mean ρ	Mean abs (ρ)
LogFSAC	−1.917 **	0.055	25.00	−0.10	0.46
LogAMLT	16.851 ***	0.000	25.00	0.87	0.87
LogCOE	18.077 ***	0.000	25.00	0.93	0.93
LogLogGIN	18.978 ***	0.000	25.00	0.98	0.98

Notes: Under the H0 of cross-section independence, CD ~ N(0,1). *p*-values close to zero indicate data are correlated across panel groups. *** and ** are the levels of significance at 1% and 5%, respectively. Source: author’s calculation (2026).

**Table 4:** Testing for slope heterogeneity.

Test	FSAC
	Statistics (*p*-Value)
Pesaran–Yamagata Delta	7.242 (0.00) ***
Pesaran–Yamagata Adjusted Delta	8.307 (0.00) ***

Note: H0: slope coefficients are homogeneous. *** The level of significance is determined at 1%. Source: author’s computation (2026).

**Table 5:** Fisher-type unit root test results.

Statistic	At a Level: Time Trend			
	LogFSAC	LogAMLT	LogCOE	LogLogGIN
P	14.45 (0.27)	136.40 (0.00) ***	12.09 (0.44)	1.88 (0.91)
Z	0.38 (0.65)	−10.16 (0.00) ***	−0.50 (0.31)	2.73 (0.10)
L*	0.09 (0.54)	−15.57 (0.00) **	−0.47 (0.32)	2.61 (0.99)
Pm	0.50(0.31)	25.39 (0.00) ***	0.02 (0.49)	−2.07 (0.98)
Statistic	At a difference: Time trend			
P	54.10 (0.00) ***	314.22 (0.00) ***	162.51(0.00) ***	79.27 (0.00) ***
Z	−5.31 (0.00) ***	−16.52(0.00) ***	−11.00 (0.00) ***	−7.31(0.00) ***
L*	−6.20 (0.00) ***	−35.91(0.00) ***	−18.57 (0.00) ***	−9.06 (0.00) ***
Pm	8.78 (0.00) ***	61.69(0.00) ***	30.72 (0.00) ***	13.73 (0.00) ***

Note: The four statistics of the Fisher test are interpreted as follows: P = inverse chi-squared; Z = inverse normal; L* = inverse logit t; Pm = modified inverse chi-squared. The values in () are *p*-values. *** and ** represent the levels of significance determined at 1% and 5%, respectively. Source: author’s computation (2026).

**Table 6:** Results of Pedroni residual cointegration test for LogFSAC model.

**a. Results of Pedroni Residual Cointegration Test for LogFSAC Model**								
**Intercept + Trend**								
H1: common AR coefficients (within-dimension)					Statistic		Prob.	
Panel v-Statistic					0.207868		0.4177	
Panel rho Statistic					1.714289		0.9568	
Panel PP-Statistic					−2.147095 ***		0.01	
Panel ADF-Statistic					−3.372625 ***		0.00	
H1: individual AR coefficients (between-dimension)								
Group rho statistic					2.554792		0.9947	
Group PP-statistic					−1.585051 **		0.0565	
Group ADF-statistic					−1.723378 **		0.0424	
b. Results of Johansen–Fisher Panel Cointegration Test								
**Hypothesized No. of CE (s)**			**Unrestricted Cointegration Rank Test**					
			Fisher Stat. * (trace test)			Fisher Stat. * (max-eigen test)		
None			62.42 (0.00) ***			33.91 (0.00) ***		
At most 1			35.96 (0.00) ***			21.45 (0.04) **		
At most 2			22.68 (0.03) **			17.11 (0.14)		
At most 3			14.64 (0.26)			14.64 (0.26)		
c. Country Cross-Section Johansen Cointegration Results								
**Hypothesized No. of CE(s)**	**No cointegration** (r = 0)		**At most 1** (r ≤ 1)		**At most 2** (r ≤ 2)		**At most 3** (r ≤ 3)	
Country	Trace	Max-Eigen	Trace	Max-Eigen	Trace	Max-Eigen	Trace	Max-Eigen
Bahrain	80.32 *** (0.00)	31.00 ** (0.06)	49.32 *** (0.01)	23.57 * (0.09)	25.75 ** (0.05)	15.65 (0.16)	10.10 (0.12)	10.10 (0.12)
Kuwait	75.57 *** (0.00)	28.13 (0.14)	47.44 *** (0.02)	18.58 (0.33)	28.86 *** (0.02)	16.49 (0.12)	12.37 ** (0.05)	12.37 ** (0.05)
Oman	69.05 *** (0.02)	29.52 (0.10)	39.54 (0.10)	19.81 (0.25)	19.73 (0.24)	15.39 (0.17)	4.33 (0.69)	4.33 (0.69)
Qatar	78.90 *** (0.00)	35.14 *** (0.02)	43.76 ** (0.04)	25.34 ** (0.05)	18.42 (0.31)	11.47 (0.46)	6.95 (0.35)	6.95 (0.35)
Saudi Arabia	66.69 *** (0.02)	26.27 (0.21)	40.4176 * (0.08)	23.23 (0.10)	17.19 (0.40)	12.3369 (0.38)	4.85 (0.61)	4.85 (0.61)
UAE	72.46 *** (0.00)	37.53 *** (0.00)	34.93 (0.24)	17.23 (0.43)	17.70 (0.36)	13.27 (0.30)	4.43 (0.67)	4.43 (0.67)

Note: a: The test used the following trend assumptions: deterministic intercept and trend, lag length selection based on AIC with a max lag of 1, Newey–West bandwidth selection, and Parzen kernel rule. b: The values in the brackets are *p*-values which are estimated using the asymptotic chi-square distribution. c: The values in the bracket are *p*-values, which are estimated using the MacKinnon–Haug–Michelis test [49]. ***, ** and * represent the levels of significance at 1%, 5%, and 10%, respectively. Source: author’s computation (2026).

**Table 7:** Posterior estimates of the random-effects model.

Variable	Mean	Std. Dev.	MCSE	Median	Equal-Tailed [95% Cred. Interval]	
					LCI	UCI
LogAMLT	−0.021209	0.0142937	0.000805	−0.021211	−0.049277	0.0068408
LogCOE	−0.377711	0.0598728	0.006129	−0.377067	−0.493342	−0.261617
LogLogGNI	5.332334	0.7855962	0.047868	5.330755	3.797512	6.863648
_cons	1.186791	0.5825959	0.017582	1.190111	0.041774	2.3197
var_U	0.0573621	0.0645225	0.005306	0.040422	0.013214	0.2015299
sigma2	0.0017601	0.0002116	2.5 × 10^−6^	0.001744	0.001393	0.002219

Note: LCI and LCI are lower and upper cred. Interval. Default priors are used for model parameters. Acceptance rate = 84%. Efficiency: min = 1%, average = 15.3% and max = 72.73%. MCMC iterations = 12,500. Source: author’s calculations (2026).

**Table 8:** Result of Bayesian mixed-effects model for LogFSAC.

Predictors	β	Std. Err.	z	P > z	[95% Conf. Interval]	
					LCI	UCI
LogAMLT	−0.0166623	0.010763	−1.55	0.12	−0.0377575	0.0044328
LogCOE	−0.3398506	0.1116245	−3.04 ***	0.00	−0.5586306	−0.1210706
LogLogGNI	4.078488	0.9175272	4.45 ***	0.00	2.280168	5.876808
_cons	2.225023	0.7191851	3.09 ***	0.00	0.8154461	3.6346
Random-effects parameters						
Country: Unstructured	Estimate	Std.err			LCI	UCI
var(LogCOE)	0.0588215	0.0714575			0.0054385	0.6361983
var(LogLogGNI)	2.334615	4.181084			0.0697895	78.09807
var(_cons)	1.642685	1.939594			0.1623682	16.61909
var(Residual)	0.0008811	0.0001091			0.0006912	0.0011232
Conditional intraclass correlation	ICC	Std. err.			LCI	UCI
	0.9994639	0.0006383			0.9944904	0.9999481
Model diagnostics: goodness of fit						
χ^2^ (6)	402.00 ***					
Log likelihood	284.25892					
Wald chi2(3)	22.72 ***					

Note: ICC is conditional on zero values of random-effects covariates. *** indicates the *p*-value is significant at the 1% level. Source: author’s calculations (2026).

**Table 9:** Posterior summary statistics of food security access in GCC countries.

U0[ID]	Mean	Std. Dev.	MCSE	Median	[95% Conf. Interval]	
					LCI	UCI
Bahrain	−0.087376	0.071238	0.017767	−0.0906652	−0.2259911	0.0493463
Kuwait	−0.003209	0.0667399	0.017486	−0.0064119	−0.1321672	0.1290095
Oman	−0.200991	0.0677622	0.017507	−0.2040084	−0.3325712	−0.0685589
Qatar	0.267964	0.0665131	0.017481	0.2635331	0.1367926	0.3994668
Saudi Arabia	0.1081877	0.0733545	0.017982	0.105241	−0.0155159	0.2493129
UAE	0.1115191	0.0667497	0.017533	0.1064313	−0.0090681	0.2448354
ID: U0:sigma2	0.0481571	0.0508716	0.001861	0.035463	0.0118455	0.1623135
e.LogFSAC: sigma2	0.0018079	0.0002649	3.3 × 10^−6^	0.0017733	0.0014078	0.002403

Note: MCMC sample size = 10,000. Source: author’s calculations (2026).

**Table 10:** Gelman–Rubin convergence diagnostic for FSAC model results.

Predictors	RC
LogLogGIN	1.001659
LogCOE	1.001355
LogAMLT	1.000879
_cons	1.002009
sigma2	1.000853

Note: Convergence rule: RC < 1.1. Source: author’s computation (2026).

**Table 11:** Bayesian posterior predictions for LogFSAC.

Prediction Horizon	LogFSAC	pmean	cri_l	cri_u
1	4.7631958	4.692064	4.613032	4.785274
2	4.7580083	4.726679	4.639222	4.812965
3	4.7411256	4.723269	4.64109	4.809749
4	4.7358535	4.734518	4.648729	4.819342
5	4.7327563	4.741408	4.658506	4.821645
6	4.7281776	4.748561	4.661359	4.830856
7	4.7217439	4.760283	4.670232	4.848299
8	4.7220726	4.727373	4.643483	4.815534
9	4.7223492	4.722018	4.635026	4.804381
10	4.7047149	4.714261	4.626261	4.793235
11	4.7050491	4.719086	4.637659	4.800364
12	4.7253586	4.735483	4.656505	4.815007
13	4.7365304	4.761668	4.678283	4.846438
14	4.7433247	4.742714	4.655333	4.833723
15	4.740882	4.740621	4.655526	4.822452

Note: pmean = posterior mean prediction; cri_l and cri_u = lower and upper bounds of the 95% credible interval. Source: author’s calculations (2026).

**Table 12:** Posterior predictive summary for model fit evaluation.

T	Mean	Std. Dev.	E(T_obs)	P(T≥T_obs)
pmin	4.516768	0.024597	4.564124	0.02
Pmax	5.157555	0.0259333	5.163135	0.38

Note: E(T_obs) = expected observed value; P(T ≥ T_obs) = the corresponding posterior predictive *p*-value; P(T≥T_obs) close to 0 or 1 indicates a lack of fit. Source: author’s computation (2026).

**Table 13:** MCMC sampling efficiency results for FSAC predictors.

Predictors	ESS	Corr. Time	Efficiency
logMET	36.51	27.39	0.0365
logCOE	17.02	58.76	0.0170
loglogGIN	44.51	22.47	0.0445
_cons	205.46	4.87	0.2055
sigma2	835.68	1.20	0.8357
var_U	1000.00	1.00	1.0000

Note: MCMC sample size is 10,000. Efficiency rate: min = 0.01702; avg = 0.3565 and max = 1%. Source: author’s computation (2026).

**Table 14:** Results of PCSE and FGLS estimators for food security access.

Predictors	PCSE Estimator				FGLS Estimator			
	β	SE.	Z	*p*-Values	β	SE.	Z	*p*-Values
AMLT	−0.011	0.0207	−0.53	−0.519	−0.011	0.0348105	−0.31	0.753
COE	−0.189	0.0760619	−2.49	0.013	−0.1891893	0.0899662	−2.10	0.035
GIN	5.084346	1.663676	3.06	0.002	5.084346	1.877766	2.71	0.007
_cons	0.4422095	1.357799	0.33	0.745	0.4422095	1.535411	0.29	0.773

Note: For the PCSE estimator, coefficients are estimated using a linear model with correlated (balanced) panels and no serial autocorrelation (Wald X^2^ = 52.91, Prob > X^2^ = 0.00). For the FGLS estimator, coefficients are estimated using generalized least squares, assuming homoscedastic panels and no serial autocorrelation (Wald X^2^ = 10.10, Prob > X^2^ = 0.0178, log-likelihood = 83.25645). Source: author’s computation (2026).

## Data Availability

The original data presented in the study are openly available in FAOSTAT at https://www.fao.org/faostat/en/#data/QCL (accessed on 15 March 2026), and World Development Indicators: World Development Indicators|DataBank (accessed on 15 March 2026).
